# How safe and effective are paediatric virtual fracture clinics? A systematic review

**DOI:** 10.3389/fdgth.2023.1261035

**Published:** 2023-10-27

**Authors:** Emma Waite, Zubair Ahmed

**Affiliations:** ^1^ College of Medical & Dental Sciences, University of Birmingham, Birmingham, United Kingdom; ^2^ Institute of Inflammation and Ageing, University of Birmingham, Birmingham, United Kingdom

**Keywords:** paediatric, child, virtual fracture clinic, telecommunication, safety, effectiveness, cost reduction, parent satisfaction

## Abstract

**Introduction:**

Virtual fracture clinics (VFC) involve a consultant-led multidisciplinary team meeting where cases are reviewed before a telephone consultation with the patient. VFCs have the advantages of reducing waiting times, outpatient appointments and time off school compared to face-to-face (F2F) fracture clinics. There has been a surge in VFC use since the COVID-19 pandemic but there are still concerns over safety in the paediatric population. Fractures make up a large burden of paediatric injuries, therefore research is required on the safety and efficacy of paediatric VFCs. This systematic review will look at the safety and effectiveness of paediatric VFCs, as well as determine the cost-effectiveness and parent preferences.

**Methods:**

As per the PRISMA guidelines two independent reviewers searched the following databases: Medline, Embase and Web of Science. Studies were included if children under 18 years old presented to A&E with a suspected or confirmed simple un-displaced fracture and were referred to a VFC. The primary outcomes assessed were effectiveness and safety, with the secondary outcomes of cost-effectiveness and parent satisfaction.

**Results:**

Six studies met the inclusion criteria for this systematic review. There was a high rate of direct discharge from the VFC leading to reduced outpatient appointments. All patients were seen within 72 h of presentation. There were limited incidences of missed fractures and the rates of re-presentation were similar to that of F2F orthopaedic clinics. There were significant cost savings for the hospitals and high parent satisfaction.

**Discussion:**

VFCs have shown to be safe and effective at managing most stable, low operative risk paediatric fractures. Safety must be ensured with a telephone helpline and an open return to fracture clinic policy. More research is needed into specific paediatric fracture types to be managed in the VFC.

**Systematic Review Registration:**

https://www.crd.york.ac.uk/PROSPERO/#searchadvanced, identifier: CRD42023423795.

## 1. Introduction

In 2011, the Glasgow fracture pathway saw the introduction of Virtual Fracture Clinics (VFC) in the management of non-operative fractures (1). This involved a consultant-led, multidisciplinary team (MDT) meeting where cases were reviewed and a management plan created before a telephone consultation with the patient. Patients were either discharged by telephone, referred to a nurse-led clinic, or referred to a specialty clinic. The VFC model has since been adopted by many other orthopaedic departments with a surge in use due to the COVID-19 pandemic (2–4).

The incidence of paediatric fractures is high, with an estimated annual incidence of 20.2 per 1,000 children in the UK, contributing to a large socioeconomic burden (5). Attendance to a face-to-face (F2F) fracture clinic generates indirect costs due to parents taking time off work, the need to organise childcare and missed schooling (6). This cost was quantified in a study by Morris and Bell, showing that every fracture clinic appointment equated to the loss of 0.25 work days, 0.18 days wages and 0.54 days of schooling (7). However, there is growing evidence that many simple undisplaced paediatric fractures require minimal, non-operative intervention (8–16). For many simple fractures, splintage and symptom management is all that is required, with minimal changes to management after a fracture clinic appointment.

In 2013, the British Orthopaedic Association Standards for Trauma (BOAST 7) guidelines were published on fracture clinic services. This stated that all acute traumatic orthopaedic injury patients should be seen within 72 h of initial presentation in a new fracture clinic (17). This target can be challenging to meet with an oversubscribed NHS and long waiting lists, resulting in patients receiving suboptimal care. However, the initiation of a VFC may enable greater compliance with this target of 72 h from presentation (18).

There are also some concerns over implementing a paediatric VFC model such as varying internet or telephone access and safeguarding issues. Furthermore, many parents may prefer a F2F appointment, resulting in re-presentation at Accident and Emergency (A&E) or General Practice (GP). There is also the potential of missing fractures in the VFC due to paediatric care often relying heavily on examination.

There is currently a need for new guidelines and research into the use of VFCs. In 2015 the BOA issued a statement on VFCs, stating that there was currently insufficient evidence to make any recommendations and welcoming more research into new fracture clinic models (19). Additionally in 2016 NICE issued new clinical guidelines on non-complex fracture management and recommended research on the clinical and cost-effectiveness of new virtual fracture clinics (20).

There have been two systematic reviews completed on VFCs, however only one of these included data on paediatrics, which was limited (21, 22). These demonstrated that VFCs can reduce waiting times, reduce costs and improve satisfaction in adult populations. Therefore, with fractures making up a huge burden of paediatric injuries (5), there is the need to look at the evidence for paediatric VFCs. This systematic review aims to look at the evidence on how safe and effective the paediatric VFC model is, as well as determine cost-effectiveness and parent preference.

## 2. Methods

### 2.1. Search strategy

The Preferred Reporting Items for Systematic Reviews and Meta-Analysis (PRISMA) statement was used for the conduction and reporting of this systematic review (23). The PICO strategy was used to define the research question and refine the search terms. Population included children under 18 years old presenting to A&E with suspected or confirmed simple undisplaced fractures. The intervention involved referral to a consultant-led VFC, with the comparison being a F2F consultant-led fracture clinic. The primary outcomes are effectiveness and safety, with the secondary outcomes of cost-effectiveness and parent satisfaction (see Table 1).

**Table 1:** Study outcomes.

Primary outcomes	Secondary outcomes
Effectiveness: Outcome after VFC; reduced outpatient appointments; Reduced X-RAYS; time to be seen	Cost-effectiveness: Cost reduction
Safety: Missed fractures; unexpected returns to GP or A&E; safeguarding; inappropriate use of VFC	Parent satisfaction: Parent preference; time off work; need to organise childcare

At the start of April 2023 a search was conducted across the following databases: Medline, Embase and Web of Science. The search terms (“Paediatric” OR “Children” OR “Child”) AND (“Virtual fracture clinic” OR “Telecommunication fracture clinic”) were used.

### 2.2. Inclusion and exclusion criteria

The inclusion and exclusion criteria were as follows. Inclusion criteria: (1) children <18 years old, (2) referral to a VFC following presentation to A&E, (3) suspected or confirmed simple undisplaced fractures and (4) English language. Exclusion criteria: (1) remote consultation for diagnosis, (2) data from VFC and F2F fracture clinics combined, (3) adult and paediatric data combined, (4) children requiring immediate orthopaedic intervention or surgery, (5) review articles, (6) case reports, and (7) conference abstracts (see Table 2).

**Table 2:** Inclusion and exclusion criteria.

Inclusion criteria	Exclusion criteria
Children <18 years old	Remote consultation on diagnosis
Suspected or confirmed simple undisplaced fractures	Data from VFC and F2F fracture clinics combined
Referral to VFC after A&E presentation	Adult and paediatric data combined
English language articles	Immediate orthopaedic input or surgery required
	Review articles, case reports and conference abstracts

### 2.3. Data extraction and synthesis

The literature search was conducted independently by two reviewers (EW and ZA). After duplicates were removed, titles were screened to exclude irrelevant studies, before abstract screening and finally full text review. If any discrepancies arose, this was resolved by discussion and mutual agreement. Identified articles underwent a systematic bibliography screen to identify further relevant articles.

A pre-designed data extraction form was used to extract data on study characteristics and outcomes. Data on author, year, location, methods, patient demographics, fracture types and patient numbers were extracted for study characteristics. The following data was extracted on outcomes: outcome after VFC, number of outpatient appointments, number of radiographs, time to be seen in clinic, incidence of missed fractures, number of unexpected returns to A&E or GP, incidences of unable to contact patients, safeguarding, inappropriate use of VFC, cost reduction, parent satisfaction, time off work and need to organise childcare.

### 2.4. Risk of bias

The quality of each study was assessed using the Risk Of Bias In Non-randomized Studies—of Exposure (ROBINS-E) tool (24). For each study seven domains including confounding, exposure measurement, participant selection, post-exposure intervention, missing data, outcome measurement and selection of reported result were assessed, and an overall risk of bias was calculated. Two independent reviewers completed this for each study (EW and ZA) with any discrepancies discussed and resolved by mutual agreement.

## 3. Results

### 3.1. Study selection

**Figure 1:**
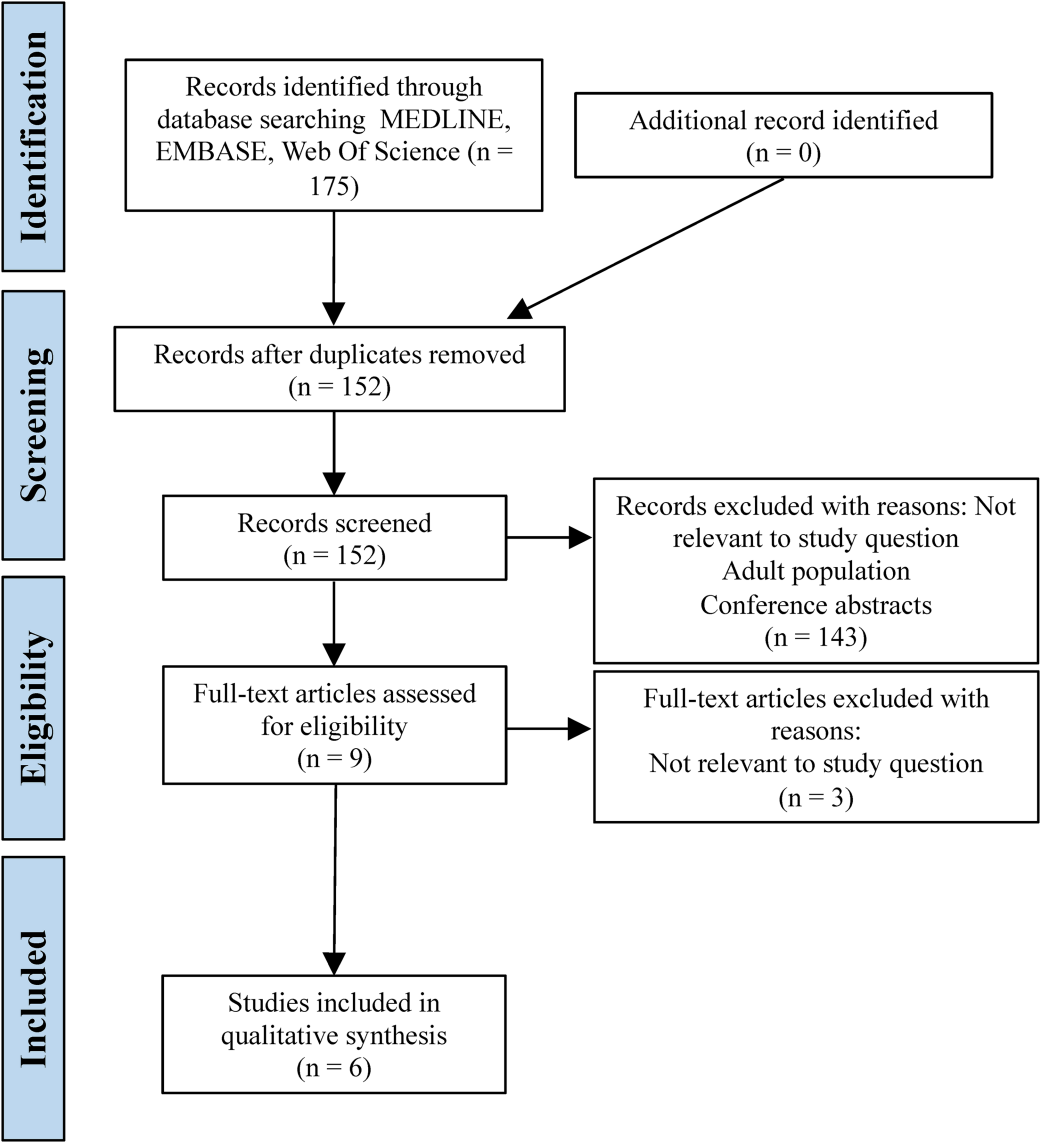
PRISMA flowchart demonstrating the literature search process.

The search strategy identified 175 articles through the databases Medline, Embase and Web of Science. 23 duplicates were removed, leaving 152 articles for screening of title and abstract. 143 articles were removed due to not assessing a VFC, paediatric and adult data combined or telecommunication used for initial diagnosis. This left nine articles for full text review; a further three were excluded due to not assessing the study question, resulting in a total of six studies to be included in the qualitative synthesis. The literature search process is summarised in a PRISMA flowchart (Figure 1).

### 3.2. Study characteristics

All six included studies were observational, with three being prospective (25–27) and three being retrospective (28–30). Three of the studies took place in Ireland (25, 27, 30) with the remaining three in England (26, 28, 29). Table three summarises the patient characteristics including mean age, sex distribution and fracture locations.

All studies displayed criteria for which fractures were suitable for the VFC and all of these included stable undisplaced or minimally displaced fractures, which were not in need of urgent orthopaedic input. The fracture locations are shown in Table 3 and all but two studies assessed a range of different fracture locations. The most common fracture, assessed by five out of the six studies, was a torus fracture of the distal radius. Torus fractures, also named buckle fractures, are incomplete injuries of long bones involving buckling of the bony cortex. The remaining study by Aboelmagd et al. assessed the use of a VFC for suspected scaphoid fractures (28) which often have minimal radiological evidence on diagnosis but have a high morbidity if missed due to the risk of avascular necrosis (31). The sex distribution was close to evenly split with a slight male predominance (25–28).

**Table 3:** Summary of study characteristics.

Study	Sample size	Mean age (years)	Sex distribution	Fracture locations (% of total)
O’Reilly et al. (25)	98	10.8	55% male 45% female	Distal radius—torus (40.8%) Metatarsal (19.4%) Metacarpal (4.1%) Finger (23.5%) Toe (12.2%)
Robinson et al. (26)	229	9.3	53% male 47% female	Hand—metacarpal & phalangeal (19.1%) Distal radius—torus (43.8%) Elbow (7.4%) Clavicle (4.3%) Metatarsal (9.3%) Lateral malleolus (16%)
Aboelmagd et al. (28)	175	13.8	65% male 35% female	Scaphoid (100%)
Kennedy et al. (27)	3,961	8.9	57% male 43% female	Hand (22%) Foot (14%) Distal radius—torus (22%) Supracondylar—Gartland 1 (13%) Lateral malleolus (10%) Clavicle (7%) Proximal humerus (4%) Toddler fracture—tibia (3%) Volar plate of finger (1%) Miscellaneous (1%)
Seewoonarain et al. (29)	44	NA	NA	Distal radius—torus (100%)
Breathnach et al. (30)	30	5.6 (clavicle) 10.6 (torus)	NA	Clavicle (33.3%) Distal radius—torus (66.6%)

### 3.3. Risk of bias

**Figure 2:**
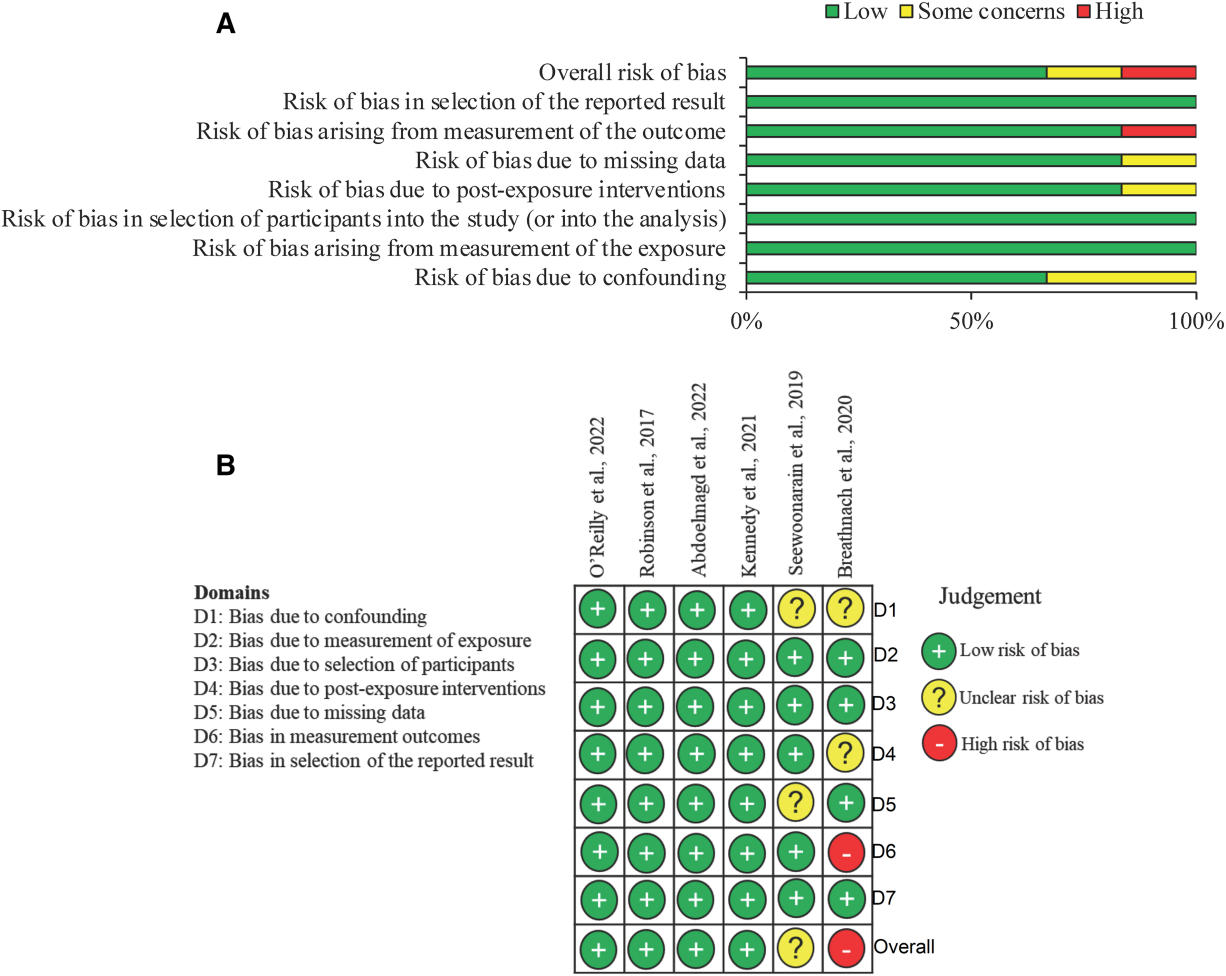
Risk of bias summary using the risk of bias in non-randomised studies—of exposure (ROBINS-E) tool. (**A**) Summary chart for the risk of bias in all studies for each domain. (**B**) Diagram to represent risk of bias in individual studies for each domain being assessed.

Using the ROBINS-E tool, the risk of bias was assessed across seven domains. Four of the studies were deemed low risk of bias in all domains (25–28). One study lacked reporting of patient demographics which could contribute to confounding factors (29). One study had a high risk of bias in measurement of outcomes as the follow up occurred after 2 years in which time post-exposure bias and error in information recall could occur (30). Combining all the studies resulted in an overall low risk of bias (66.7%). Figure 2 summarises the risk of bias across all individual studies and the combined risk.

### 3.4. Statistical analyses

Due to the heterogeneity in the reporting, a narrative summary of the studies is presented with a summary of the main study characteristics tabulated. No additional quantitative data synthesis was performed.

### 3.5. Primary outcomes

#### 3.5.1. Effectiveness

Three studies reported on outcomes after the VFC. The rate of direct discharge from VFC without the need for a further appointment ranged from 70% to 75% (27–29). This resulted in the saving of the following number of F2F fracture clinic appointments: 78/104 (28), 2,770/3,961 (27) and 33/44 (29). Robinson et al. estimate that three VFC patients can be seen in the time it takes to review one F2F fracture clinic patient (26). Additionally, Seewoonarain et al. reported a 94% reduction in the number of repeat radiographs after a VFC model was introduced for torus fracture management (29).

Five of the studies reported on the time between initial presentation and review in the VFC and all of these were within 72 h, indicating high compliance with the BOAST 7 guidelines (25, 27–30).

#### 3.5.2. Safety

Two studies reported the incidence of missed fractures at the VFC. One reported no missed fractures during the study period (28) and one reported a missed scaphoid fracture which was picked up when the patient returned due to ongoing pain (26).

Five studies reported on unexpected patient returns to A&E, GP, or the fracture clinic through an open return policy. Of those discharged from the VFC, unexpected re-presentations ranged from 0% to 6.6% (25, 28–30). The main reasons for this were parental concern, ongoing pain and re-injury as shown in Table 4.

**Table 4:** Reasons for re-presentation and percentages.

Reason for re-presentation	Percentage of total
Parental/carer concern	49.7%
Ongoing pain	35.8%
Re-injury	7.3%
Primary care referral	2.1%
Change of dressing or orthosis	1.0%
Investigation of limp	1.0%
Paraesthesia	0.5%
Wound issue	0.5%
Other/unknown	2.1%

Four studies reported clinician unfamiliarity with the VFC pathway, leading to the incorrect use of the VFC protocol. This resulted in patients referred to the F2F fracture clinic that would have been suitable for the VFC (26, 28–30). The reported numbers range from 22.4% to 48.4% of patients who were seen in the F2F fracture clinic but were suitable for the VFC, therefore additional appointments could have been saved. The most common deviation from the protocol was application of plaster of Paris in A&E instead of a removable soft cast. This resulted in an additional hospital appointment to remove the plaster, compared to a soft cast which can be removed at home. Four of the studies reported this occuring (26, 28–30). Despite the initial unfamiliarity with the VFC protocol, Kennedy et al. reported that over time the number of patients referred to the VFC and the numbers discharged from the VFC increased. This indicates increased clinician confidence with the pathway over time (27). Only one report of safeguarding was mentioned by Aboelmagd et al, where one family refused to engage with the pathway and were uncontactable by phone, initiating the safeguarding process (28). They reported that 97% of families were contactable by phone.

### 3.6. Secondary outcomes

#### 3.6.1. Cost-effectiveness

Four of the studies reported a cost reduction with the VFC model compared to the F2F fracture clinic (26–29). Robinson et al. reported an estimated annual combined saving of £106,000 for the hospital and Clinical Commissioning Group (CCG) with the implementation of their VFC pathway, which involved reviewing 21 virtual patients a week. They reported a saving of £21 per torus fracture patient managed by the VFC pathway. Aboelmagd et al. reported a total saving of £8,814 in the study period due to 78 outpatient appointments saved, resulting in a saving of £113 per patient virtually discharged. Seewoonarain et al. reported a saving of £61.22 per torus fracture patient managed in the VFC compared to standard practise. Kennedy et al. worked out the cost saving in the Irish health care system reporting a saving of €101 per patient and a NET saving of €254,120 in the study period of 2 years and 7 months. Significant cost savings both for the hospital and CCG were shown to be achieved with the paediatric VFC model compared to the F2F fracture clinic.

#### 3.6.2. Parent satisfaction

Two studies assessed parent or guardian satisfaction with the VFC pathway (25, 30). The reported parental satisfaction was high with 96.9% and 100% of parents strongly agreeing or agreeing that they were satisfied with their child's recovery. Additionally, Robinson et al. reported a low request for F2F appointments indicating satisfaction with the virtual pathway.

O'Reilly et al. reported that 41.8% of parents or carers would need to organise childcare in order to attend a F2F fracture clinic appointment. Moreover, Breathnach et al. reported 70% of parents or carers would need to organise additional childcare and 87% would have to take time off work to attend a F2F fracture clinic.

## 4. Discussion

### 4.1. Effectiveness

Studies have shown that a significant number of paediatric fracture clinic referrals are inappropriate and could be reduced by as much as 30% (32, 33). In this review, paediatric VFCs were associated with a high rate of direct discharge, as much as 75%, resulting in reduced outpatient appointments and radiographs. Reducing the burden on the outpatient department results in shorter waiting times and more appointments available for specialist cases. Furthermore, all studies in this review showed high compliance with the BOAST 7 guidelines, enabling patients to be seen in a consultant-led VFC within 72 h of initial presentation (17). This has also been shown with the adult VFC model, with significantly reduced waiting times and patients seen within 72 h (18, 21, 22). By reaching this target, patient care is optimised and trauma care is standardised.

### 4.2. Safety

With the paediatric VFC model, there may be concern over missing fractures due to the inability to examine the child. However, there were minimal incidences of missed fractures reported in this review. There was only one report of a missed scaphoid fracture which was picked up after 15 days of unsettling pain (26). This does not differ from standard practice, where repeat imaging is performed between 2 and 6 weeks due to the low sensitivity of plain radiographs in excluding scaphoid fractures. To improve safety and reduce the chances of missing fractures, strict inclusion and exclusion criteria of who to refer to the VFC is vital. This should include the ages of the children as well as injury locations of fracture types that typically heal well with minimal intervention. Additional safety netting procedures should be put in place so that the child can return to clinic or seek advice if required. Three studies reported an open return to clinic policy (26, 28, 30) where patients could return to the fracture clinic if any issues occurred. Five of the studies reported that a telephone advice line was available for direct communication with the fracture clinic (25–28, 30). These ensure that in the case of further complications, help can be sought quickly.

The rate of unexpected re-presentations after VFC discharge remained comparable to the rates of unplanned readmissions in orthopaedic and paediatric trauma specialties. The rates of unplanned re-presentations in the review were 0%, 2.2%, 2.6%, 3% and 6.6% respectively (25–28, 30). Bernatz et al. found the unplanned readmission rate across orthopaedic subspecialties was 3% (34), while Wheeler et al. found a 1.7% unplanned readmission rate in paediatric trauma patients (35). The greatest cause of re-presentation in this review was parental/carer concern. This signifies the importance of reassurance and adequate information giving to parents on the home management of their child's injury. Home care advice leaflets specific to the child's fracture type are beneficial, as well as a telephone contact if reassurance or advice is required.

One issue highlighted in this review was clinician unfamiliarity with the VFC protocol. Large numbers of patients were referred to the F2F clinic who could have been referred to the VFC. There were found to be decreased referrals to the VFC around the times that orthopaedic trainee changeovers occurred (27) indicating that this was due to inexperience with the VFC model. Therefore, educating clinicians on the VFC protocol and specific guidelines on which fracture types and ages are suitable for VFC referral is vital. Kennedy et al. reported a positive correlation between VFC referrals and subsequent patient discharge over time, indicating that as clinicians became more familiar with the protocol, their confidence using it increased (27).

A further concern with the paediatric VFC model is the risk of missing safeguarding opportunities in children with fractures. There was only one report of a safeguarding incidence in this review, with Aboelmagd et al. mentioning a family who were not contactable by phone causing initiation of the safeguarding process (28). Several studies reported methods of reducing the safeguarding risk such as excluding children under 18 months from the VFC pathway (26). It has been shown that 80% of paediatric fractures due to abuse occur in children under 18 months (36), so by ensuring these patients are seen in a F2F clinic, the risk can be reduced. Furthermore, if parents refuse to engage with the VFC pathway or there is any suspicion of abuse in A&E, then the safeguarding process should be initiated.

### 4.3. Cost-effectiveness

In terms of cost-effectiveness, the studies in this review show a significant cost reduction for the hospital and CCG with the implementation of paediatric VFCs. Robinson et al. estimate a saving of £10.1 million per year if all NHS hospitals in England implemented VFCs, based on 2016 clinic data (26). Evidence of this significant cost saving has also been shown in adult VFC populations (21, 22). Direct cost savings to the hospital are due to the lower cost of soft splints compared to hard casts, reduced outpatient appointments, reduced radiographs and shorter waiting times freeing up appointments for specialist cases. Furthermore, there are many indirect costs related to attending a F2F fracture clinic appointment. Holm et al. estimate that the indirect cost of attending one paediatric fracture outpatient appointment, due to loss of productivity, is €78.4 per consultation (6). VFC implementation would allow less time off work for parents, fewer wages lost, fewer childcare costs, as well as less time out of school for children (7). Therefore, the total cost savings of VFCs are likely greater than calculated.

### 4.4. Parent satisfaction

The reported parent or carer satisfaction with the VFC model was high, despite a small population sampled (125 parents). There were large numbers of parents that would need to take time off work or organise further childcare in order to attend a F2F fracture clinic appointment, indicating preference for the virtual clinic. Parental satisfaction can be improved with good communication and information giving as well as clear sign posting to the fracture clinic telephone advice lines.

### 4.5. Limitations and scope of the VFC model

One limitation of the VFC model is difficulty in communicating between the orthopaedic department and A&E. As displayed in four of the studies, miscommunication led to several patients placed in plaster of Paris hard casts instead of soft, removable casts. This meant they required a further trip to hospital to remove this when they could have been discharged virtually. Therefore, good communication between orthopaedic and A&E departments is vital, including shared guidelines.

A further limitation of VFCs is that they are only suitable for certain stable, low operative risk fractures, meaning there may be limited application of the VFC. Nevertheless, this review has shown that many simple undisplaced paediatric fractures can be reviewed in a VFC as long as there is safety netting with an open return to fracture clinic policy and telephone advice line. The fracture types managed by a VFC in this review included: metatarsal, toe, metacarpal, finger, torus (distal radius), elbow, clavicle, lateral malleolus, scaphoid, Gartland 1 supracondylar, proximal humerus and toddlers' fracture of the tibia. The evidence for managing specific paediatric fracture types in the VFC is limited. Table 5 summarises the available evidence for managing three paediatric fracture types in the VFC model.

**Table 5:** Evidence for specific paediatric fracture types managed in the VFC.

Fracture type	Evidence	Management
Torus	There has been much evidence supporting the minimalistic approach to managing torus fractures (10, 12, 15)	Immobilise with soft cast or splint in A&E and discharge in VFC
	Soft casts and splints have led to higher patient and parental satisfaction compared to rigid casts. Rigid casts cause greater pain and result in a longer time to return to normal activities (14–16)	
	NICE guidelines advocate the use of soft casts or splints rather than rigid casts (20)	
	Studies in this review displayed the effectiveness of managing torus fractures in a VFC with high rates of direct discharge, high parental satisfaction and reduced costs (25–27, 29, 30)	
Clavicle	There is evidence supporting the non-operative management of undisplaced paediatric clavicle fractures, with minimal need for follow up (13, 37)	Undisplaced paediatric clavicle fractures can be managed with a broad arm sling or collar & cuff in A&E and discharged in VFC
	A retrospective study over 25 years showed no difference in outcomes whether managed operatively or conservatively (38)	
	Studies in this review demonstrated high rates of clavicular fracture discharge through the VFC, with high parental satisfaction, reduced need for outpatient appointments and reduced cost (26, 27, 30)	
Scaphoid	Aboelmagd et al. demonstrated a pathway for managing suspected paediatric scaphoid fractures in the VFC (28). This pathway resulted in reduced outpatient appointments and reduced costs. There were no missed fractures on short term follow up	Immobilise suspected scaphoid fracture in futuro splint. If no fracture is shown on initial radiograph, repeat radiograph within 2 weeks. Discharge in VFC if no fracture shown on repeat imaging
		Referral to outpatient orthopaedic appointment if scaphoid fracture is found

## 5. Limitations

This systematic review is subject to several limitations which are related to the quality of the included studies. The studies were all observational, limiting the strength of the evidence, and only English language studies were included which may contribute towards selection bias. Additionally, heterogeneity in the included studies such as different health care systems, fracture types and methods of fracture immobilisation may limit the drawn conclusions from the data. The studies provided limited data on the immobilisation methods and treatment of specific fracture types, meaning few recommendations on specific fracture types could be made. Furthermore, the majority of the research is published by those directly involved in setting up a VFC, meaning the research may be subject to publication bias. Finally, evidence may be limited by small sample sizes in certain outcomes groups such as parental satisfaction.

## 6. Conclusion

VFCs have shown to be an effective way of managing most stable, low operative risk paediatric fractures, as long as adequate parental guidance is given and the opportunity to contact the clinic is provided. There were high rates of direct discharge from the VFC, without the need of a further F2F review saving many outpatient appointments and reducing the need for repeat radiographs. Additionally, waiting times were reduced, with all patients seen within 72 h in line with the current BOAST 7 guidelines. The VFC model appears safe with low rates of re-presentation along with minimal incidences of missed fractures. VFC implementation was associated with a significant cost reduction compared to the standard F2F fracture clinic model, resulting in savings both for the health care system and patients. Finally, there was a high rate of parental satisfaction with the model, leading to less time off work and more time in school for children. However, there is limited evidence available on specific paediatric fracture types managed in the VFC. More research is required to improve the management of specific paediatric fracture types in the VFC.

## Data Availability

The original contributions presented in the study are included in the article/Supplementary Material, further inquiries can be directed to the corresponding author.
